# Under-Reported Aspects of Platinum Drug Pharmacology

**DOI:** 10.3390/molecules22030382

**Published:** 2017-02-28

**Authors:** Dirk Theile

**Affiliations:** Department of Clinical Pharmacology and Pharmacoepidemiology, University of Heidelberg, Im Neuenheimer Feld 410, 69120 Heidelberg, Germany; dirk.theile@med.uni-heidelberg.de; Tel.: +49-6221-5639-400

**Keywords:** cisplatin, pharmacology, mechanism of action, immunogenic cell death, RNA, toxicity

## Abstract

Platinum drugs remain the backbone of many antineoplastic regimens. Among the numerous chemical or pharmacological effects of platinum drugs, some aspects tend to be under-reported. Thus, this perspective paper intends to stress some neglected properties of platinum drugs: first, the physico-chemical characteristics (aquation reaction kinetics) that determine site-specific toxicity; second, the impact on RNA molecules. Knowledge of the ‘RNA world’ has dramatically changed our understanding of cellular and molecular biology. The inherent RNA-crosslinking properties should make platinum-based drugs interact with coding and non-coding RNAs. Third, we will discuss the impact on the immune system, which is now recognized to substantially contribute to chemotherapy efficacy. Together, platinum drugs are in fact old drugs, but are worth re-focusing on. Many aspects are still mysterious but can pave the way to new drugs or an improved application of the already existing compounds.

## 1. Introduction

Cisplatin, oxaliplatin, and carboplatin belong to the most prominent antineoplastic drugs used against a broad set of solid malignancies. Their physico-chemical properties, molecular pharmacology, and clinical efficacies or toxicities have been described in great detail. However, even four decades after cisplatin’s approval, there are still debatable aspects of platinum drug pharmacology. To date, some parts of platinum drugs’ pharmacodynamics are still poorly characterized. Moreover, there are known effects and properties of platinum drugs that are hardly highlighted in the scientific literature. Most—but not all [1]—review articles concentrate on the DNA platination-mediated effects (e.g., induction of apoptosis, inhibition of transcription or replication, etc.), with good cause, because the structural and functional disturbances of genomic or mitochondrial DNA seem to be at the bottom of platinum drug pharmacology (both efficacy and adverse side effects). However, there are aspects that are constantly under-reported. Admittedly, the relevance of such by-stander effects is not entirely known, but chronically ignoring them harbors the potential that advancements in metal-based drugs (especially platinum drugs) are missed out on or hugely delayed.

Thus, this perspective paper tries to complete the long list of data on the specific properties of platinum-based drugs. It also intends to remind the scientific community of platinum drug characteristics that might pave the way to a final understanding of platinum drugs’ unique efficacies and toxicities, respectively.

## 2. Impact of Platinum Drugs’ Physico-Chemical Properties on Site-Specific Toxicities

**Figure 1:**
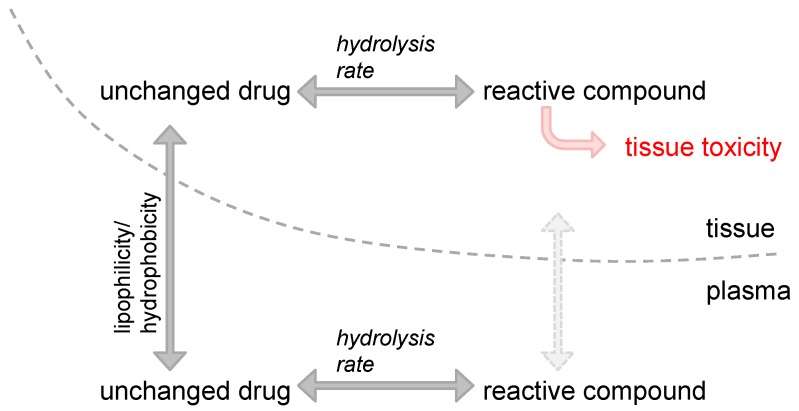
Impact of hydrolysis rate (aquation reaction kinetics) on tissue distribution and toxicity. Tissue distribution of unchanged platinum drug is mainly determined by the lipophilicity or hydrophobicity of its carrier and leaving group, respectively. After substitution with water, the positively charged reactive compound present in plasma (and potentially bound to plasma proteins) can hardly penetrate tissue barriers. In contrast, reactive compounds present in the tissue will lead to toxicity.

All clinically used platinum drugs have a central platinum(II) atom and thus the same basic structural make-up and mode of activation. After losing the respective leaving group and subsequent substitution with water, the positively charged molecules are nucleophilically targeted by the free electrons of the N7 atom of purines to form intrastrand and interstrand adducts [2]. Despite this fundamental conformity, the platinum drugs differ with respect to systemic and cellular pharmacodynamics (concentration-effect relationship), pharmacokinetics (relationship between time and concentration at a certain site), and toxicity [3,4,5]. These differences are based on the physico-chemical properties of the platinum drugs’ carriers and leaving groups, respectively. Cisplatin was modified to carboplatin by changing the chloride ligands to a dicarboxycyclobutane ring, which markedly altered the tissue distribution pattern [6], systemic pharmacokinetics [3], and toxicity profile [5]. While cisplatin is highly nephro- and ototoxic, carboplatin is mainly burdened by myelosuppression [5]. Intuitively, one would try to explain such site-specific toxicities by the pharmacokinetics of the platinum drug itself. That means one would look at either the systemic exposure to the parent drug or to the tissue-specific concentration-time profile of the drug (distribution characteristics), respectively. Indeed, carboplatin distributes differently than cisplatin [6]. However, it seems that site-specific toxicity is not only a question of the pure distribution of the unchanged drug but is rather determined by three main factors: first, the plasma kinetics (mainly the area under the curve, AUC_p_), which is directly related to the dose (when applied intravenously); second, the plasma-tissue concentration ratio, indicating the capacity of the drug to penetrate tissue; and third, the rate of platinum drug activation through ligand substitution with water. In a landmark study, the relationships between these parameters were used to predict (or model) the nephrotoxicity and myelosuppression of cisplatin, carboplatin, and nedaplatin in rats [7]. After administering platinum drugs to male Wistar rats, the linearity of pharmacokinetics, the total clearance and the apparent ratio of tissue concentrations of the unchanged drug to the plasma concentration (Kp_app_) at steady state were determined. The apparent hydrolysis rates of each drug were concurrently determined in vitro. Nephrotoxicity and myelosuppression were estimated by blood urea nitrogen (BUN) and platelet count, respectively. Tissue exposure to the active platinum compound was estimated as the product of the AUC_p_ of the unchanged drug and a ‘toxicity factor’. This ‘toxicity factor’ in turn is the product of Kp_app_ and the apparent hydrolysis rate constant k_hydrolysis_. Interestingly, the BUN levels (= nephrotoxicity) differed significantly among the compounds, even at the same exposures (mg·Pt·mL^−1^·min^−1^). The inclines of the BUN levels also differed, being very steep with cisplatin and almost nonexistent with carboplatin. So, it is hard to estimate what actually determines tissue toxicity. However, when the activation kinetics were considered (represented in the ‘toxicity factor’), the interrelations cleared up: the relationship between the AUC_p_ × ‘toxicity factor’ and BUN did fit well to an Emax model. In bone marrow, this function was also correlated with the platelet count, a surrogate for myelosuppression. Together, the product of AUC_p_ × ‘toxicity factor’ was shown to determine the pharmacokinetics/toxicodynamics relationship of platinum drug–induced nephrotoxicity and myelosuppression in rats. In summary, this study impressively demonstrated the tremendous (clinical) importance of the physico-chemical properties of platinum drugs or their respective leaving groups. It moreover reminds us that neither the dose, nor the plasma exposure (AUC_p_) or tissue exposure to the parent compound determines toxicity, but that activation kinetics (k_hydrolysis_) matters since it is the active compound of a drug that causes pharmacological (side) effects (Figure 1). This also suggests that through prudent selection of respective leaving groups, platinum drugs can be designed to exhibit particular pharmacological characteristics.

## 3. Interaction of Platinum Drugs with RNA

DNA is considered the primary target of platinum drugs. Many of the numerous downstream effects begin with structural disturbances of DNA [8]. Thus, it makes sense to expect that cytotoxicity is tightly correlated to the degree of DNA platination (e.g., pg platinum per µg of DNA). However, some experimental studies challenged this assumption. When different cancer cell lines were exposed to cisplatin or oxaliplatin, two interesting findings were obtained [9]: First, the correlation between DNA platination and cytotoxic potency (IC_50_ value) was rather vague. Second, oxaliplatin caused considerably fewer DNA adducts, but its potency was higher or at least comparable. Together, this data questions whether DNA is the only or exclusive pharmacological target of platinum drugs. In consequence, the relevance of other macromolecules needs to be evaluated. Proteins have in fact been investigated and characterized as platinum drug targets, but in less detail. It is at least well known that cisplatin, oxaliplatin, and carboplatin differ in their protein-binding affinities and that proteins with reduced thiol groups show a high affinity for platinum drugs [10]. It has been demonstrated that platinated proteins can lose their function (ubiquitin: [11]; heat shock proteins: [12]; microtubules: [13]) and do not set the platinum drug free again to re-locate to nucleotides [14].

**Figure 2:**
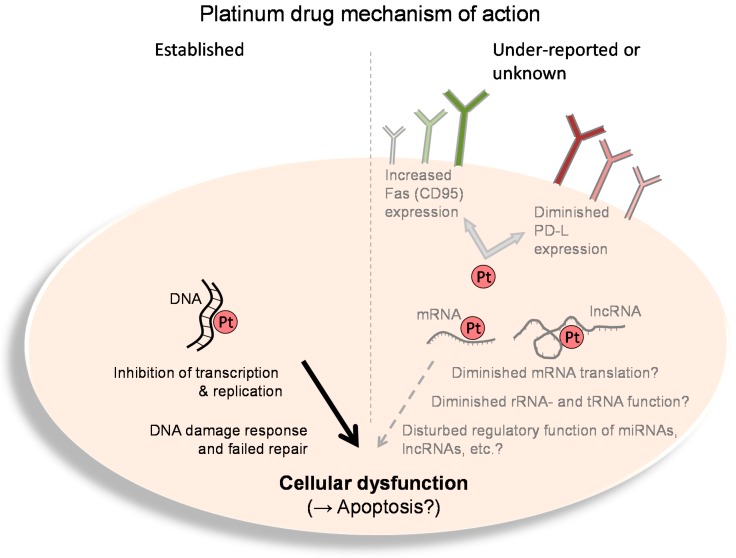
Platinum drug mechanism of action. Left: The established and well-known mechanisms of action of platinum drugs (Pt) comprise platination of genomic and mitochondrial DNA. This leads to hindrance of DNA-based functions (e.g., transcription, replication, etc.). Such cellular dysfunction eventually causes apoptosis, especially when DNA lesions cannot be repaired. Right: The under-reported or currently unknown effects of platinum drugs (Pt) comprise functional inhibition of diverse RNA species such as messenger (m)RNA, ribosomal (r)RNA, transfer (t)RNA, micro(mi)RNA, long non-coding (lnc) RNA, etc. Moreover, platinum drugs such as cisplatin can cause upregulation of Fas (CD95) or downregulation of programmed death ligand (PF-L) expression, eventually leading to enhanced immunosurveillance or T cell–mediated cell lysis, respectively.

The third important biological macromolecule has, however, been poorly characterized as a platinum drug target: RNA. Well-performed experimental analyses have found platinum adducts in both ribosomal (r)RNA [15] and transfer (t)RNA [16]. Moreover, it was even demonstrated that RNA is quantitatively and kinetically preferred over DNA to be targeted by platinum drugs [17,18], which can be partly attributed to the high negative charge density found in complex RNA molecules compared to the rather linear form of DNA [17]. Together, these findings imply that platinum drugs could lead to diminished protein translation through disturbance of ribosome function, eventually depleting (tumor) cells of essential proteins (Figure 2). Whether such platinum adducts at rRNA or tRNA truly contribute to cisplatin’s anti-cancer efficacy or toxicity is, however, still under debate. Indeed, functionally relevant rRNA sequences were shown to be platinated by cisplatin, but most experimental studies used drug concentrations never achieved in vivo [17,19]. Recently, another experimental study tried to evaluate the impact of platinum drugs at clinically relevant concentrations on translation efficiency in vitro. Using an adenocarcinoma cell line (LS180 cells) transfected with a vector encoding a destabilized green fluorescent protein (GFP) version with a short half-life, no relevant effect of cisplatin, oxaliplatin or carboplatin on GFP expression (=translation efficiency) was documented [20]. Since the positive control cycloheximide also exhibited only minor effects, the authors concluded that cells or tissues with a high(er) protein turn-over might still be susceptible to translation inhibition caused by rRNA or tRNA platination.

Platinum-mediated adducts were also detected in messenger (m)RNA [17]. Using a cell-free approach (not biased by cellular background) interaction of cisplatin with mRNA has recently been demonstrated to hinder mRNA translation. Exposing GFP-encoding mRNA to platinum drugs for 1 h, cisplatin (and oxaliplatin) was shown to concentration-dependently inhibit GFP protein synthesis, whereas carboplatin had no effect [21]. It is likely that the lack of the carboplatin effect was due to its 100-fold slower ligand substitution (=activation) kinetics [22,23], making the 1 h exposure too short to cause functionally relevant RNA lesions.

Drug-mediated disturbance of RNA structure or function can nevertheless contribute to the cytotoxic effect of many antineoplastic agents (anti-metabolites, anthracyclines), because RNA insults were suggested to also induce apoptosis [24]. RNA’s relevance for drug cytotoxicity is furthermore underlined by clear evidence that there are special cellular mechanisms for coping with RNA lesions. First, AlkB and hABH3 (two DNA demethylases) can repair methyl methanesulfonate–mediated methylation of bacteriophage MS2 RNA in vivo [25]. Second, there are pleiotropic proteins that are implicated in various mechanisms of DNA damage response that could also play a role in the damage of RNA. For instance, YB-1 is a protein with a chaperone and transcription factor function that recognizes damaged nucleic acids and helps to repair or dispose them [26]. YB-1 has so far been mostly connected with the DNA damage response, but there is very good data showing that YB-1 selectively binds to 8-oxoguanine–modified RNA but scarcely to non-modified RNA [27]. This suggests that YB-1 might modulate a cell’s fate after exposure to RNA-damaging agents such as platinum drugs. A few studies have in fact connected YB-1 expression with chemotherapy resistance and poor prognosis in cancer [28,29], but it still remains unclear whether this is truly connected to YB-1’s role as a potential RNA damage control protein or to its function as a transcription factor influencing drug resistance and cell cycle genes [26,29]. Together, there is nevertheless enough data advocating for the presence of cellular mechanisms (proteins, response pathways) that can recognize and process RNA damages such as RNA platination.

RNA platination could also fine-tune the huge cellular impact of non-coding RNAs (Figure 2). However, there is no definite data about the impact of platinum drugs on the cellular function of endogenous microRNAs or long non-coding RNAs, although such RNA species were recently suggested as potential drug targets (antisense oligonucleotides, aptamers, ribozymes) [30]. However, experimental studies have at least evaluated the influence of cisplatin and oxaliplatin on the silencing capacity of exogenous small-interfering RNAs (siRNAs). After treating the antisense strand, the platination resulted in adducts with protection against hydrolytic cleavage in the proximity of the platination sites rendering the respective siRNA more stable. Expression assays confirmed the conserved biological activity of antisense-platinated siRNAs. However, the capacity of silencing was reduced through platination. Of the two complexes studied, oxaliplatin exhibited the larger influence, thus indicating subtle differences between the drugs to interfere with si- and maybe miRNA processing [31].

In summary, recent evidence clearly shows that RNA is targeted by platinum drugs. However, it is currently hard to estimate whether RNA platination is of any in vivo relevance. Effects were in fact observed, but most experimental studies were either cell-free approaches [21], used non-mammalian cells [17], exposed cells to drug concentrations never achieved in vivo [17], or failed to record relevant functional effects [20]. In regards to the RNA damage response, it has been difficult to distinguish whether observed effects are RNA damage–specific or a by-stander effect that accompanies the process of DNA damage response. Prospectively, it seems worth deeply characterizing the RNAs that are platinated after (clinical) administration of the compounds. For instance, evaluating whether specific mRNAs, microRNAs or long non-coding RNAs of the inner ear are platinated might help to further scrutinize the mechanism behind cisplatin-mediated ototoxicity. In addition, investigating RNA platination–mediated induction of apoptosis or searching for potential RNA damage response pathways would definitely expand our knowledge on RNA-platinum drug interactions.

## 4. Immunogenic Effects of Platinum Drugs

As underlined in the previous sections, current evidence for the mode of action of platinum drugs concentrates on the platination of molecules (DNA, RNA) present in the target tumor cell. However, recent evidence shows that the anti-cancer efficacy of platinum drugs is not restricted to effects triggered in the tumor cell. In contrast, immunological aspects seem to play a major of role (Figure 2). In fact, most classic antineoplastic agents are regarded as immunosuppressive, but accumulating evidence indicates that the innate and adaptive immune system largely contributes to the anti-cancer effect of conventional chemotherapies. For instance, since the 1980s, cyclophosphamide has been known to stimulate the anti-tumor actions of the immune system. Cisplatin has also been recognized to exert effects that boost immunosurveillance. When human colon carcinoma cell lines were exposed to cisplatin, this treatment enhanced ICAM-1 and Fas (= CD95) expression, resulting in antigen-specific cytotoxic T-lymphocyte (CTL)-mediated lysis involving Fas-dependent and -independent mechanisms [32]. Moreover, cisplatin is known to enhance T cell activation by dendritic cells through the dephosphorylation of signal transducer and activator of transcription (STAT) 6 [33]. This lack of STAT 6 phosphorylation then leads to diminished transcription of STAT 6 target genes such as programmed death ligand (PD-L) 2. Eventually, the T cell–inhibitory effect of PD-L2 is accordingly lowered and T cell activity (or activation) is enhanced. The described mechanism of STAT6 inactivation seems to be of clinical significance. STAT 6 overexpressing head and neck cancers show poor outcome compared to non-overexpressing tumors. However, when STAT 6 overexpressing cancers were treated with radiotherapy and cisplatin (leading to STAT 6 dephosphorylation), STAT 6 overexpression seemed to be beneficial [34]. This data shows that cisplatin can trigger immunological effects of clinical relevance. Besides the T cell–stimulating effects, platinum drugs can specifically enhance immunogenic cell death (ICD) [35]. Today, three mechanisms leading to ICD have been thoroughly characterized [35]: first, the release of ATP by apoptotic cells as a ‘find-me’ signal for dendritic cells and macrophages subsequently infiltrating the tumor; second, calreticulin exposure at the surface of cancer cells as an ‘eat-me’ signal for dendritic cells; third, release of high mobility group box 1 (HMGB-1) protein from the nucleus leading to pro-inflammatory cytokine secretion. Cisplatin causes the release of ATP and HMGB-1, thus fulfilling two of the three prerequisites for ICD. However, because cisplatin does not provoke calreticulin exposure in tumor cells [36], cisplatin does not induce full-blown ICD. Since cisplatin is frequently combined with ICD-inducing radiotherapy or cytostatics, respectively, ICD can nevertheless occur with cisplatin administration. Together, the immunological effects of cisplatin (and other platinum drugs) are well characterized, but should be further evaluated, because this aspect of platinum pharmacology harbors the potential for improvements in clinical application of (immune-)chemotherapy. This is underlined by experimental and clinical data suggesting a considerable synergism between the immune-stimulatory effects of platinum drugs and checkpoint inhibitors (antibodies against CTL antigen 4, PD-1 or PD-L1) [35,37].

## 5. Conclusions

Among the numerous pharmacological effects of platinum drugs, some aspects tend to be under-reported: first, the make-up of the leaving group and the implicated kinetics of the aquation reaction. This easily overlooked physico-chemical detail has a huge impact on biochemical and clinical aspects. Through prudent design of respective leaving groups, it should be possible to synthesize platinum drugs with specific desired characteristics. Second, the impact on RNA molecules has been under-reported. Since the early 1990s, the ‘RNA world’ has dramatically changed our understanding of cellular and molecular biology. Profound evidence indicates that platinum drugs significantly interact with the numerous RNA species. Due to their inherent RNA-crosslinking properties, platinum drugs (and other metal-based drugs such as ruthenium derivatives) must be expected to diversely interact with coding and non-coding RNAs. Third, the impact on the immune system has been under-reported. Immunological contributions to chemotherapy efficacy are becoming more and more known among oncologists and are a modern aspect of pharmacological research. Although platinum drugs have been established for decades, it is worth re-focusing on these compounds because many aspects are poorly understood or have the potential to pave the way to new drugs or an improved application of the already existing compounds.
